# Six Cases of Tumors

**Published:** 1890-01

**Authors:** Daniel D. Lee

**Affiliations:** Instructor in Anatomy, Veterinary School of Harvard University


### SIX CASES OF TUMORS.

By Daniel D. Lee, M.D.V.,
*Instructor in Anatomy, Veterinary School of Harvard University.*

*Case I.*—In the latter part of October, I was called to see a grey gelding, seven years old, who had suffered from a severe attack of febra pyogenica in the early summer, from which he had never entirely recovered. He was in fair condition, but had no appetite, and when driven had paroxysms of violent and distressing coughing. On inquiry, I found that the owner, being unable to find a veterinarian at the place where he was spending the summer, had treated the animal himself. The symptoms that the horse had exhibited, were described to me, and I had no doubt that he had had an attack of febra pyogenica, which had run its usual course. I examined the horse and found the temperature to be 102°·F, the mucous membranes pale and the pulse weak. The respiration was like that of a horse suffering from heaves, but not accelerated. When made to cough, a small amount of tenacious mucous was expelled. The skin and tissues of the intermaxillary space were thickened from the effect of the abscesses. There was no nasal discharge, except when coughing. Examination of the lungs by Dr. Peters showed a large area of dullness in the right ung, and a small area in the left. Taking into consideration the history of the case, the constant elevation of temperature, and the long time that the cough had been present, three months, the diagnosis was made that small abscesses had formed in the lungs by metastasis. That the abscesses had set up chronic interstitial pneumonia, which had encysted the abscesses and caused the consolidation of the lung tissue. I told the owner that I did not think that the horse would ever recover from the cough, but that he might live for a long time. As the horse was young and much valued, I attempted treatment at the owner's request. After a month of tonic and stimulating treatment, great attention being given to the food, the condition of the horse was very good, with the exception that the cough was just as bad, and that the temperature was constantly elevated from $101\frac{3}{5}$° to 102° F. In the latter part of December, it being impossible to drive the horse because of his cough, by my advice he was killed.

The post-mortem examination showed in the right lung two large, round, fibrous tumors, very dense and hard, and about five inches in diameter. In the left lung, one small tumor of the same character, but only about two inches in diameter. On section these tumors, which were sharply defined from the healthy lung tissue, proved to be due to an hypertrophy of the interstitial tissue of the lung. The bronchial tubes traversing these tumors were pressed upon by the increased interstitial tissue, so that on section they resembled narrow slits in the lung tissue. This pressure on the bronchial tubes had caused a local chronic bronchitis which accounted for the cough. Dr. Whitney, of the Harvard Medical School, made a microscopical examination of portions of the diseased lung, which confirmed my diagnosis. With the exception of the lungs the organs were all normal. The thickening in the intermaxillary space had entirely disappeared under the influence of a blister.

*Case II.*—A large St. Bernard dog, very much emaciated, and at the time suffering from acute gastritis. The attack of gastritis was cured, but the stomach remained very weak, and unless very carefully fed, all food would be thrown up. A week or two afterwards a complete atrophy of the muscles on the left side of the head took place, to such an extent that nothing but the skin covered the bone, the eye sunk in and the appearance of the dog was exceedingly unpleasant. Mastication was accomplished with great difficulty, as only one side of the jaw was strong enough. Later, swallowing became very difficult and this was found on examination to be due to a collection of swellings in the region of the throat. Other swellings appeared in the axilla and groin, and the dog went lame. I killed him, and at the post-mortem found, besides the complete atrophy of the muscles of the left side of the head, diffuse lymphomata of the entire body. I can best describe the condition by saying that every lymphatic gland in the body was enlarged, varying from a pea to an egg in size. The largest of these tumors were found in the throat, axilla, groin, and about the thoracic and abdominal viscera. This accounted for the difficulty in swallowing, lameness and difficulty of retaining food.

The atrophy of the muscles of the side of the head was probably caused by the pressure of some of the tumors interfering with the nutrition of the part. The spleen besides being the seat of several distinct lymphomata, was so greatly enlarged that it weighed eighteen ounces.

*Case III.*—An aged white horse was given to me. He had an enormous melantoric tumor involving the penis and sheath. This hung down eight inches, and when the horse trotted, flapped from side to side. I cast the horse and turned him on his back ; after some difficulty I found the meatus urinarius, hidden in the mass of the tumor, and passed the catheter. I then cut down into the catheter just under the arch of the ischism, and laying open the urethra for three inches, sewed its edges to those of the divided skin. By three rapid cuts, I dissected out the entire penis and sheath in front of the opening, leaving only the stalk of the penis, around which I passed five strong elastic ligatures. The hæmorrhage was trifling. I then allowed the horse to rise, and three days after removed the mass, which was cold and dead. The wound was two months in healing, and the horse is now working.

*Case IV.*—A favorite horse of my own had a polypoid wart, as large as a man's fist, on the end of the penis, near the meatus. I cast him and removed it, and on microscopical examination it was at first pronounced to be cancer, but later the diagnosis of non-malignant papillary fibroma was made. Within a year I was obliged to remove the growth four times, but finally I succeeded in extricating it, and the horse, previously always thin, is now in fine condition.

*Case V.*—I was called to see a spaniel dog who had a large soft tumor involving the base of the tail and the parts around the anus. The tumor was so large as to seriously deform the dog. I etherized the dog and removed the growth which proved to be a lipoma. Although I had to dissect the skin off the tail for three-quarters of the way around, yet a complete recovery without any scar resulted.

*Case VI.*—A bay horse, driven in a milk wagon, started out from the barn at an early hour and never drove better. After being driven for an hour-and-a-half he suddenly fell in the shafts. He was unharnessed and taken to a stable where I found him in a very excited state, unable to control his motions, staggering, and falling with legs spread out, getting up and going through the same performance. He made several attempts to drink but could not swallow. He died eight hours after in acute frenzy.

Post-mortem examination showed a tumor of the pituitary body of the brain a little larger than a pigeon's egg. This was situated just behind the optic chiasma. The horse had never shown any symptoms of brain trouble before, and had been used by the same person several years.

